# [Retracted] Prostaglandin E2 reduces swine myocardial ischemia reperfusion injury via increased endothelial nitric oxide synthase and vascular endothelial growth factor expression levels

**DOI:** 10.3892/br.2026.2116

**Published:** 2026-02-02

**Authors:** Ying Zhou, Peng Yang, Aili Li, Xiaojun Ye, Shiyan Ren, Xianlun Li

Biomed Rep 6:188–194, 2017; DOI: 10.3892/br.2016.834

Following the publication of the above paper, it was drawn to the Editor’s attention by a concerned reader that the images in Fig. 2A-D on p. 190, showing staining of the myocardium, had already appeared in three other publications (in the journals *Journal of Zhejiang University SCIENCE B* in 2011, *Chinese Journal of Integrative Medicine* in 2012, and *Artificial Cells, Blood Substitutes, and Biotechnology* in 2012) that were either submitted by the same research group, or featured some of the same authors. Given that these data had not been credited as having appeared in print prior to the receipt of this paper at *Biomedical Reports*, the authors were asked to provide an explanation. In their response, they indicated that the images featured in all papers were intended to show the same data, using the same experimental set-up (and that, for these papers, different authors within the research group had selected the same representative images, which reflected the average typical findings). In addition, the authors mentioned that the drug used in this study was prostaglandin E1 (PGE1), although it was mistakenly referred to as PGE2 in the paper, and that this oversight should not have affected the published results.

Owing to the fact that the abovementioned data had already been published prior to its submission to *Biomedical Reports,* the Editor has decided that this paper should be retracted from the Journal. Upon receiving this decision from the Editor, the authors did not accept the decision that the article be retracted. The Editor apologizes to the readership for any inconvenience caused.

